# Impact of solid lipid nanoparticles on 3T3 fibroblasts viability and lipid profile: The effect of curcumin and resveratrol loading

**DOI:** 10.1002/jat.4379

**Published:** 2022-08-26

**Authors:** Antonella Rosa, Mariella Nieddu, Giulia Pitzanti, Rosa Pireddu, Francesco Lai, Maria Cristina Cardia

**Affiliations:** ^1^ Department of Biomedical Science University of Cagliari Cagliari Italy; ^2^ Department of Life and Environmental Sciences University of Cagliari Cagliari Italy

**Keywords:** cytotoxicity, fibroblasts, lipid profile modulation, nanoparticles, SLN

## Abstract

This study focused on the impact in 3T3 fibroblasts of several types of empty and curcumin‐ and resveratrol‐loaded solid lipid nanoparticles (SLN) on cell viability and lipid metabolism in relation to their lipid content and encapsulated drug. SLN, prepared by hot homogenization/ultrasonication, were characterized with respect to size, polydispersity index, and zeta potential. Compritol® 888 ATO at different concentrations (4%, 5%, and 6% wt/wt) was chosen as lipid matrix while Poloxamer 188 (from 2.2% to 3.3% wt/wt) and Transcutol (TRC; 2% or 4%) were added as nanoparticle excipients. Prepared SLN were able to encapsulate high drug amount (encapsulation efficiency percentage of about 97–99%). All empty SLN did not show cytotoxicity (by MTT assay, at 24 h of incubation) in 3T3 cells independently of the lipid and TRC amount, while a viability reduction in the range 5–11% and 12–27% was observed in 3T3 cells treated with curcumin‐loaded and resveratrol‐loaded SLN, respectively. SLN without TRC did not affect cell lipid metabolism, independently from the lipid content. Empty and loaded SLN formulated with 4% of Compritol and 4% of TRC significantly affected, after 24 h of incubation at the dose of 5 μl/ml, cell polar lipids (phospholipids and free cholesterol) and fatty acid profile, with respect to control cells. Loaded compounds significantly modulated the impact of the corresponding empty formulation on cell lipids. Therefore, the combined impact on lipid metabolism of SLN and loaded drug should be taken in consideration in the evaluation of the toxicity, potential application, and therapeutic effects of new formulations.

## Abstract 2

Empty, curcumin‐loaded, and resveratrol‐loaded SLN, prepared with Compritol® 888 ATO and Transcutol by hot homogenization/ultrasonication, were characterized for size, polydispersity index, and zeta potential and were tested in 3T3 fibroblasts. Empty nanoparticles were not cytotoxic at 24 h of incubation, while a 5–11% and 12–27% cell viability reduction was observed for curcumin‐loaded and resveratrol‐loaded nanoparticles, respectively. Empty and loaded SLN with 4% Compritol/4% Transcutol affected (5 μl/ml) cell lipid profile, and the impact on lipid metabolism was strictly modulated by loaded compound.

## 1 INTRODUCTION

Lipids (triacylglycerides, phosphoglycerides, sterols, and sphingolipids) play several important roles at cellular and organismal levels, having structural, energy, signaling, and immunoregulatory functions (Denisenko et al., 2020; Santos & Schulze, 2012). Fatty acids (FA) are the main building blocks of numerous lipid species and contribute to several complex biochemical processes (Ferreri et al., 2016; Santos & Schulze, 2012). FA, as structural components of membranes and inflammation/anti‐inflammatory mediators, display regulatory effects on cell homeostasis and physiological functions and represent indispensable substrates for β‐oxidation and ATP production (Ferreri et al., 2016; Maulucci et al., 2016). Following the biosynthesis, desaturase enzymes introduce a methylene group in saturated FA (SFA) leading to the monounsaturated FA (MUFA), while polyunsaturated FA (n‐6 and n‐3 PUFA) are produced by elongation and desaturation of the essential linoleic (18:2 n‐6) and α‐linolenic (18:3 n‐3) acids, respectively (Ferreri et al., 2016; Maulucci et al., 2016). Free cholesterol, together with phospholipids, is an essential component in the plasma membrane of mammalian cells and plays diverse structural and functional roles (Paukner et al., 2022). Changes in lipid organization (FA and cholesterol) can largely affect membrane functional and biophysical properties (fluidity and lipid rafts organization), severely altering cellular functions such as membrane trafficking, protein dynamics, and signal transduction (Ferreri et al., 2016; Maulucci et al., 2016; Paukner et al., 2022; Santos & Schulze, 2012). These membrane‐related effects can cause disease in living organisms (Paukner et al., 2022). FA profile has been considered as a homeostatic and metabolic biomarker in normal and pathological cells (Maulucci et al., 2016). Recent studies have proposed FA ratios as a suitable way to efficiently replace the original FA data set for FA metabolism analysis (Graeve & Greenacre, 2020). Cholesterol and/or FA metabolism represent the target for the treatment of several pathological conditions such as metabolic syndrome (Denisenko et al., 2020), diabetes (Langlois et al., 2021), and cancer (Santos & Schulze, 2012). Targeting altered lipid metabolic pathways (FA biosynthesis and desaturation, phospholipids and cholesterol metabolism, and lipid droplet synthesis) has become a promising anticancer strategy (Liu et al., 2017; Rosa et al., 2019; Santos & Schulze, 2012). Moreover FA, in a free form or incorporated into complex lipids, are crucial to proper functions of the epidermis and its appendages (Lin & Khnykin, 2014), and the alteration of skin lipid composition can lead to several dermatological disorders (Drakou et al., 2021). Several phenolic compounds act through the membranes by interacting with membrane lipids and changing the general lipid membrane biophysical properties (structure, organization, fluidity, and packing), membrane dynamics, and the expression of lipogenic enzymes involved in FA metabolism (Kühn et al., 2018; Reis & de Freitas, 2020; Rosa et al., 2020).

Nanoparticles, a class of functional materials with overall dimensions in the nanoscale range, are amply used in pharmacology and medicine (Dhiman et al., 2021; Faraji & Wipf, 2009). Lipid‐based nanoparticles (LN), made from biocompatible and biodegradable lipids, are well tolerated in living systems, and represent important systems for enhanced incorporation of hydrophobic compounds into the lipid matrix (Dhiman et al., 2021; Pizzol et al., 2014; Severino et al., 2012). Among these nanostructures, solid lipid nanoparticles (SLN), characterized for presenting a solid lipid matrix at room temperature and body temperatures, have been extensively used in pharmaceutical and cosmetic formulations (Faraji & Wipf, 2009; Pizzol et al., 2014). SLN are generally regarded as nontoxic, biocompatible, and easy‐to‐produce formulations and are widely being explored for improving dermal/transdermal and oral delivery of drugs, nutraceuticals, and cosmeceuticals (Dhiman et al., 2021; Faraji & Wipf, 2009; Musielak et al., 2022; Pizzol et al., 2014; Severino et al., 2012). Particularly, SLN main molecular constituents are physiologically compatible lipids (FA, glycerides or other FA esters, sterols, sterol esters, waxes, etc.) and surfactants commonly used as emulsifying agent and food additive (Dhiman et al., 2021; Pizzol et al., 2014; Scioli Montoto et al., 2020).

Plainly, safe, and effective utilization of LN and SLN requires that they do not result in an adverse biological response (Scioli Montoto et al., 2020). LN and SLN cannot be viewed as a simple delivery system but can play an active role in mediating biological effects, affecting viability and cell physiology, in particular lipid metabolism (Falchi et al., 2015; Pitzanti et al., 2020; Rosa et al., 2015). Numerous articles were devoted to exploring the LN and SLN cytotoxic effects (Acevedo‐Morantes et al., 2013; Pizzol et al., 2014; Scioli Montoto et al., 2020; Weyenberg et al., 2007) and up‐regulation of pro‐inflammatory cytokines (Schöler et al., 2002) in cell systems. Previous studies evidenced the toxicity in mouse 3T3 fibroblasts, J774 macrophages, HaCaT keratinocytes, and red blood cells of SLN formulated with stearic acid (Pizzol et al., 2014; Weyenberg et al., 2007). Remarkably, only a small amount of research was undertaken to investigate the LN and SLN impact on the cell lipid profile (in terms of FA and lipid classes) (Falchi et al., 2015; Pitzanti et al., 2020; Rosa et al., 2015). Previous studies evidenced changes in lipid components (incorporation of oleic acid 18:1 n‐9 in the phospholipid and triacylglycerol fractions and lipid droplets accumulation) occurring in human carcinoma HeLa cells when exposed to short‐term treatments with monoolein‐based cubosomes stabilized by Pluronic F108 (Falchi et al., 2015; Rosa et al., 2015). Moreover, in a preliminary study, we evidenced the effect of SLN loaded with 8‐methoxypsoralen in modulating FA and polar lipid profile in fibroblasts, without affecting cell viability (Pitzanti et al., 2020). Therefore, LN should not be considered only as simple carriers for drug delivery, but also as a platform able to modulate specific lipid biosynthetic pathways (Falchi et al., 2015; Rosa et al., 2015).

**FIGURE 1:**
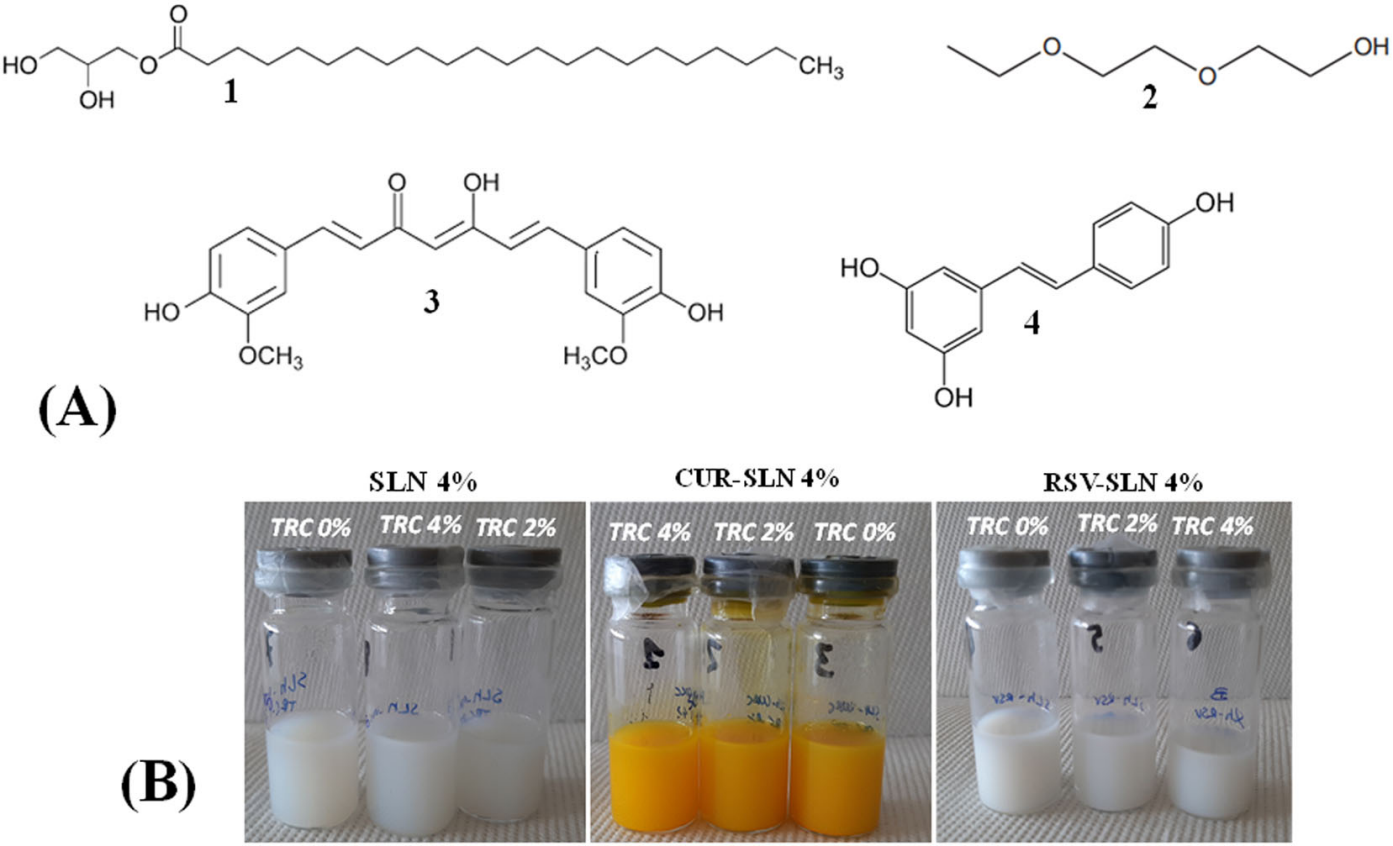
(A) Chemical structures of glyceryl behenate (1), Transcutol® P (TRC, 2), curcumin (CUR, 3), and resveratrol (RSV, 4). (B) Digital images of freshly prepared unloaded solid lipid nanoparticles (SLN) and CUR‐ or RSV‐loaded SLN with different amounts of Transcutol® P (TRC; 0%, 2%, and 4%). [Colour figure can be viewed at wileyonlinelibrary.com]

Curcumin (CUR) and resveratrol (RSV) (Figure 1A) are bioactive liposoluble polyphenolic compounds (Goel et al., 2008; Rosa et al., 2014). The dietary polyphenol CUR has been reported to possess a variety of biological and pharmacological activities (anti‐inflammatory, antimicrobial, anticarcinogenic, and antioxidant properties). The natural polyphenol RSV is consumed worldwide as food items and possesses safety profiles with diverse biological and pharmacological actions, including antioxidation, anti‐inflammation, antidiabetic, and anticancer activity (Gomes et al., 2020; Gumireddy et al., 2019). Because of their low water solubility, several studies have been devoted to improving CUR and RSV bioavailability by incorporation in different formulations (liposomes, nanosuspensions, SLN, and nanostructured lipid carriers) (Gumireddy et al., 2019; Sakellari et al., 2021). Moreover, previous studies evidenced the ability of CUR and RSV to affect cell membrane fluidity and FA metabolism (Kühn et al., 2018; Naeini et al., 2019).

In addition to effects on cell viability, the interaction of SLN with cell lipids is an essential focus in assessing and understanding their toxicity and compatibility, because SLN‐induced changes in cell lipids can influence biological membrane properties and affect cellular processes. Starting from all these considerations, the aim of this study was to evaluate the simultaneous impact of different types of unloaded and loaded SLN on cell viability and lipid metabolism in relation to their composition (lipid content, penetration enhancer, and encapsulated compounds). SLN were prepared with different amounts of Compritol® 888 ATO (monoesters, diesters, and triesters of behenic acid 22:0; the structure of glyceryl behenate is reported in Figure 1A) as lipid matrix, Poloxamer 188 (P188) as nanoparticle stabilizer, and Transcutol® P (TRC) (Figure 1A) as penetration enhancer, excipients amply used in cosmetic and pharmaceutical products for their tolerability (Björklund et al., 2016; Cortés et al., 2021; Devi & Agarwal, 2019). In a previous study, we demonstrated that the use of TRC in a SLN formulation containing 4% of Compritol 888 ATO could enhance the cellular uptake of nanoparticles and cell lipid modulation (Pitzanti et al., 2020). CUR and RSV were chosen as loaded compounds for their high liposolubility, bioactivity, and ability to affect lipid metabolism. Prepared SLN were tested in 3T3 fibroblasts, a normal cell line previously used to assess the toxicity and biocompatibility of empty and loaded SLN (Pitzanti et al., 2020; Pizzol et al., 2014; Weyenberg et al., 2007). Moreover, the mouse fibroblast plasma membrane is considered an excellent model system to study how FA influence the membrane (Ibarguren et al., 2014). The effect of unloaded, CUR‐loaded, and RSV‐loaded SLN (at 24 h of incubation) on 3T3 cell viability was evaluated by the MTT assay. Changes in lipid components occurring in SLN‐treated 3T3 fibroblasts (24 h of incubation) were explored monitoring cell phospholipids (PL), free cholesterol (FC), FA composition, and FA ratio profile.

## 2 MATERIALS AND METHODS

### 2.1 Materials

Pluronic F68 (Poloxamer 188, P188), CUR, RSV, standards of FA, cholesterol, 3‐(4,5‐dimethylthiazol‐2‐yl)‐2,5‐diphenyltetrazolium bromide (MTT), 1,2‐dipalmitoyl‐sn‐glycero‐3‐phosphocholine (PC 16:0/16:0), 1,2‐dioleoyl‐sn‐glycero‐3‐phosphocholine (PC 18:1/18:1), 1‐palmitoyl‐2‐oleoyl‐sn‐glycero‐3‐phosphocholine (PC 16:0/18:1), 1‐oleoyl‐2‐palmitoyl‐sn‐glycero‐3‐phosphocholine (PC 18:1/16:0), 2‐linoleoyl‐1‐palmitoyl‐sn‐glycero‐3‐phosphocholine (PC 16:0/18:2), 2‐arachidonoyl‐1‐palmitoyl‐sn‐glycero‐3‐phosphocholine (PC 16:0/20:4), 1,2‐dilinoleoyl‐sn‐glycero‐3‐phosphocholine (PC 18:2/18:2), 1,2‐dieicosapentaenoyl‐sn‐glycero‐3‐phosphocholine (PC 20:5/20:5), and all solvents (purity > 98%) were purchased from Sigma‐Aldrich (Milan, Italy). Compritol® 888 ATO (esters of behenic acid) and Transcutol® P (TRC) (Figure 1A) were kindly supplied by Gattefossè. Cell culture materials were purchased from Invitrogen (Milan, Italy). All the other chemicals used in this study were of analytical grade.

### 2.2 SLN preparation

SLN (Table 1 and Figure 1B) were prepared by a hot homogenization technique followed by ultrasonication using Compritol® 888 ATO as lipid matrix, P188 as nanoparticle stabilizer, TRC as penetration enhancer, and CUR or RSV as encapsulated compounds. The concentrations of the different components in SLN formulations were chosen on the basis of a preliminary screening (preformulation study) that had been setup to find the optimized concentration for each component (data not shown) and our previous study (Pitzanti et al., 2020) that allowed to individuate the amount of the different components that assured the higher encapsulation efficiency and long‐term physical stability of SLN (Compritol 888 ATO 4%, TRC 0–4%, P188 2.2%, encapsulated compound 0.1%). The concentration of 0.1% was selected for both RSV and CUR because no separation of the polyphenols from the lipid matrix was detected at the light microscope during 48 h of testing (data not shown). The increase of the RSV or CUR concentration determined a decrease of both the encapsulation efficiency and the total amount of loaded drugs.

**TABLE 1:** Composition of unloaded (empty) and CUR‐ or RSV‐loaded SLN prepared with different amounts of Compritol® 888 ATO (4%, 5%, and 6% wt/wt) and Transcutol® P (TRC; 0%, 2%, and 4% wt/wt)

Formulations	Compritol 888 ATO (% wt/wt)	P188 (% wt/wt)	CUR (% wt/wt)	RSV (% wt/wt)	TRC (% wt/wt)	Water (% wt/wt)
Empty‐TRC 0%‐SLN 4%	4	2.2	—	—	—	93.8
Empty‐TRC 0%‐SLN 5%	5	2.75	—	—	—	92.25
Empty‐TRC 0%‐SLN 6%	6	3.3	—	—	—	90.7
Empty‐TRC 2%‐SLN 4%	4	2.2	—	—	2	91.8
Empty‐TRC 4%‐SLN 4%	4	2.2	—	—	4	89.8
CUR‐TRC 0%‐SLN 4%	4	2.2	0.1	—	—	93.7
CUR‐TRC 2%‐SLN 4%	4	2.2	0.1	—	2	91.7
CUR‐TRC 4%‐SLN 4%	4	2.2	0.1	—	4	89.7
RSV‐TRC 0%‐SLN 4%	4	2.2	—	0.1	—	93.7
RSV‐TRC 2%‐SLN 4%	4	2.2	—	0.1	2	91.7
RSV‐TRC 4%‐SLN 4%	4	2.2	—	0.1	4	89.7

Abbreviations: CUR, curcumin; RSV, resveratrol; SLN, solid lipid nanoparticles.

Empty SLN were obtained by melting different amounts of Compritol 888 ATO (4%, 5%, and 6% wt/wt of the final SLN dispersion) at 85°C, and thereafter, a hot aqueous solution containing P188 (from 2.2% to 3.3% wt/wt of the final SLN dispersion, lipid/P188 ratio of 1:0.55) and TRC (0%, 2%, or 4% wt/wt of the final SLN dispersion) was added. The pre‐emulsion was homogenized using a high shear homogenizer (Ultra Turrax T25) and then sonicated with a Soniprep 150 (MSE Crowley, London, UK) thermostated at 85°C (50 cycles, 5 s “on”, 2 s “off”). The obtained O/W nanoemulsion was then cooled down to room temperature to produce the SLN trough lipid re‐crystallization. To prepare the SLN loaded with the two polyphenols, CUR or RSV (0.1% wt/wt of the final SLN dispersion) were dissolved in the melted lipid phase (Compritol 888 ATO at 4% wt/wt of the final SLN dispersion) before mixing with the P188/TRC hot aqueous solution. The formulations obtained were stored at 25°C prior to use and characterized 24 h after preparation.

### 2.3 SLN characterization

Average diameter (Z‐AVE) and polydispersity index (PI, as a measure of the size distribution width) of the samples were determined by Dynamic Light Scattering (DLS) using a Zetasizer nano (Malvern Instrument, Worcestershire, UK). Samples were backscattered by a helium–neon laser (633 nm) at an angle of 173° and a constant temperature of 25°C. Zeta potential was estimated using the Zetasizer nano by means of the M3‐PALS (phase‐analysis light scattering) technique. Just before the analysis, all formulations were diluted with distilled water. Encapsulation efficiency (EE%), expressed as the percentage of the drug entrapped in the lipid matrix respect to the amount of CUR or RSV initially used in the SLN preparation, was determined as follows: 1 ml of CUR‐SLN or RSV‐SLN dispersions was centrifuged at 2000 rpm for 10 min at 20°C (Scilogex D3024R, Rocky Hill, CT, USA), to separate the insoluble and non‐encapsulated drug crystals (pellet) from the SLN loading the encapsulated drug (supernatant). There were 100 μl of supernatant or unpurified SLN dispersions diluted with 900 μl CH_3_OH to extract the drug from the lipid matrix. This suspension was then centrifuged (15,000 rpm, 30 min, 4°C) to separate the lipid matrix from the supernatant containing the extracted drug. The CUR amount in the supernatant was assayed by fluorescence, using a Synergy 4 multiplate reader (BioTek, Winooski, USA) at an excitation/emission wavelength of 480/520 nm. The RSV amount was determined with the same instrument, measuring the absorbance of the supernatant at the wavelength of 306 nm by the same. Before the analysis, the stock standard solutions of CUR and RSV (0.5 mg/ml) were prepared by dissolving the drug in CH_3_OH. A standard calibration curve (fluorescence for CUR or absorbance for RSV vs. known drug concentration) was built up by using standard solutions prepared by the dilution of the stock standard solution with CH_3_OH (0.005–0.1 mg/ml). Calibration graphs were plotted according to the linear regression analysis, which gave a correlation coefficient value (*R* ^2^) of 0.998. Moreover, empty SLN were analyzed as blank.

The encapsulation efficiency (EE %) of the SLN was calculated using the following equation:

EE%=(drug concentration after purification/drug concentration before purification)×100

### 2.4 Cell culture

Mouse 3T3 fibroblasts have been obtained from the American Type Culture Collection (ATCC, Rockville, MD, USA). Cells were grown in Dulbecco's modified Eagle's medium (DMEM) with high glucose, supplemented with 2 mM L‐glutamine, penicillin (100 units/ml)–streptomycin (100 μg/ml), and fetal calf serum (FCS) (10% v/v), at 37°C in a 5% CO_2_ incubator. Subcultures of 3T3 cells were grown in T‐75 culture flasks and passaged with a trypsin–EDTA solution.

### 2.5 Experimental design for cell treatment

Preliminarily, a series of unloaded SLN formulated with different amounts (4%, 5%, and 6% wt/wt) of Compritol 888 ATO was tested in murine fibroblasts for the evaluation of cell cytotoxicity and lipid profile modulation in relation to their lipid amount to individuate the most biocompatible formulation useful for drug encapsulation. Then a series of SLN prepared with 4% wt/wt of the lipid matrix, different percentages of Transcutol® P (0, 2, 4% wt/wt), and loaded with CUR and RSV were assessed for cytotoxicity and lipid profile modulation in 3T3 to study whether the nature of the loaded compound could affect the impact of SLN on cells viability and lipid metabolism.

### 2.6 Cytotoxic activity (MTT assay)

Cytotoxicity of SLN was evaluated in 3T3 fibroblasts by the MTT assay (Pitzanti et al., 2020; Rosa et al., 2015). 3T3 cells were seeded in 96‐well plates (density of 3 × 10^4^ cells/well) in 100 μl of serum‐containing media. Experiments were carried out 2 days after seeding (at 90% cell confluence). Fibroblasts were treated for 2 and 24 h with different concentrations (1.25, 2.5, and 5 μl/ml, corresponding to 1:800, 1:400, and 1:200 v/v) of SLN formulations in 100 μl of complete fresh medium. We previously used the same concentrations of nanoparticle formulations to evaluate the effect on cell viability of monoolein‐based cubosomes stabilized by Pluronic F108 (Falchi et al., 2015; Rosa et al., 2015) and 8‐methoxypsoralen‐loaded SLN formulated with Compritol 888 ATO (Pitzanti et al., 2020). After incubation, cells were subjected to the MTT test as reported (Pitzanti et al., 2020; Rosa et al., 2015). Color development was measured at 570 nm with an Infinite 200 auto microplate reader (Infinite 200, Tecan, Austria). The absorbance is proportional to the number of viable cells, and results are shown as percentage of cell viability in comparison with control (non‐treated) 3T3 cells. The cytotoxicity of different amounts (1.25, 2.5, and 5 μg/ml) of pure CUR and RSV, from a 1 mg/ml DMSO solution, was also evaluated in 3T3 cells (after 24 h of incubation) for comparison, representing the corresponding drug amount present in the SLN tested concentrations.

A preliminary study of SLN cytotoxicity after 2 h of incubation highlighted a decrease of viability in fibroblasts treated with empty SLN, maybe due to a variation in the cell redox status that affects mitochondrial metabolization of MTT, as previously observed at short time of incubation with LN (Falchi et al., 2015). Therefore, 24‐h incubation time was chosen for successive studies in 3T3 cells.

### 2.7 Lipid profile modulation in 3T3 cells

3T3 fibroblasts were seeded in T‐75 culture flasks at a density of about 10^6^ cells/10 ml of complete medium and were used for FA profile modulation experiments at 2 days post‐seeding (90% cell confluence). 3T3 fibroblasts were treated with SLN (at a concentration of 5 μl/ml) in fresh medium and incubated at 37°C for 24 h. After treatment, cells were washed with phosphate‐buffered saline (PBS), scraped, and centrifuged for 5 min at 1200 *g* at 4°C. Cell pellets were then separated from supernatants and used for lipid extraction and analyses (Rosa et al., 2015).

### 2.8 Extraction and preparation of cell lipid components

Total lipids were extracted from 3T3 cell pellets by the addition of the mixture CHCl_3_/CH_3_OH/H_2_O 2:1:1 as previously reported (Rosa et al., 2015). Aliquots of the CHCl_3_ fraction from each cell sample, containing cell liposoluble compounds, were dried down, dissolved in CH_3_OH, and directly injected into the liquid chromatograph (high‐performance liquid chromatography [HPLC]) for the analysis of lipid components (PL and FC) (Rosa et al., 2015, 2019) and CUR. Another aliquot of the CHCl_3_ fraction was subjected to mild saponification for FA separation as reported (Rosa et al., 2015, 2019).

### 2.9 HPLC analysis of cell lipid compounds and CUR

Analyses of lipid compounds and CUR extracted from 3T3 cell pellets were carried out with an Agilent Technologies 1100 HPLC (Palo Alto, CA, USA) equipped with a diode array detector (DAD) and an Agilent Technologies 1260 Infinity ELSD (HPLC‐DAD/evaporative light scattering detector [ELSD] system). Separation of PL and FC in cell lipid extracts was performed with an Inertsil ODS‐2 column and CH_3_OH as the mobile phase, at a flow rate of 0.7 ml/min, as previously reported (Rosa et al., 2015, 2019). The same chromatographic conditions were used for the analysis of CUR, detected at 450 nm with DAD. Analyses of unsaturated (detected at 200 nm with DAD) and saturated (ELSD detection) free FA, obtained from cell lipid saponification, were carried out with a XDB–C_18_ Eclipse column equipped with a Zorbax XDB–C_18_ Eclipse guard column (Agilent Technologies), with a mobile phase of 75% of acetonitrile and 25% of acetic acid (0.5%) in water, at a flow rate of 2.3 ml/min (Rosa et al., 2015, 2019). The Agilent OpenLAB Chromatography data system was used for the recording and integration of the chromatogram data. The identification of cell lipid compounds and CUR was made using standard compounds and conventional UV spectra. Calibration curves of all compounds were constructed using standards and were found to be linear (DAD) and quadratic (ELSD), with correlation coefficients >0.995 (Rosa et al., 2015, 2019). The calibration curve equation, limit of detection (LOD), and correlation coefficient (*R* ^2^) of the individual FA standards are provided in Table S1.

### 2.10 Statistical analysis

Data were expressed as a mean ± standard deviation (*SD*). Evaluation of statistical significance of differences was performed using GraphPad InStat software (GraphPad software, San Diego, CA, USA) and with the software package XLStatistic for Excel. Multiple comparison of means groups was assessed by one‐way analysis of variance (one‐way ANOVA) followed by the Bonferroni multiple comparisons test or by post‐hoc Tukey honestly significant difference (HSD) test to substantiate statistical differences between groups, while Student's *t*‐test was used to compare two samples. Significance was tested at the 0.05 level of probability (*p*).

## 3 RESULTS

### 3.1 SLN characterization

In this study, different empty (unloaded), CUR‐loaded, and RSV‐loaded SLN formulations (Table 1) were prepared by a hot homogenization technique followed by ultrasonication. The concentrations of the different components in SLN formulations were chosen on the basis of a preformulation study (data not shown) and our previous research (Pitzanti et al., 2020) that allowed to individuate the optimized concentration for each component and the formulation that assured the SLN long‐term physical stability and highest encapsulation efficiency (Compritol 888 ATO 4%, Transcutol 0–4%, P188 2.2%, encapsulated compound 0.1%). In this work, SLN with different amounts of Compritol 888 ATO (4%, 5%, and 6% wt/wt) have been prepared to evaluate the influence of the lipid matrix concentration on the cell viability and lipid metabolism. P188 and TRC (2% or 4% wt/wt) were added as nanoparticle stabilizer and penetration enhancer, respectively. The lipid/stabilizer ratio was kept constant at 1:0.55 w/w, according to our previous research (Pitzanti et al., 2020).

Empty, CUR‐loaded or RSV‐loaded SLN formulations were deeply characterized with respect to size (average diameter), polydispersity index (PI), zeta potential (ZP), and encapsulation efficiency percentage (EE%) (Table 2). Physicochemical measurements of empty formulations without TRC (Table 2) showed that increased Compritol concentration leads to an evident growth of nanoparticle size. A statistically significant difference (*p* < 0.01) was observed for the size of empty‐TRC 0%‐SLN 6% (162.2 nm) versus empty‐TRC 0%‐SLN 4% (132.9 nm), while no significant difference was observed for empty‐TRC 0%‐SLN 5% (140.4 nm) versus 4%. Furthermore, the higher the Compritol concentration, the higher the PI values measured, thus indicating a decrease in size distribution homogeneity (0.27 for empty‐TRC 0%‐SLN 6% vs 0.24 for empty‐TRC 0%‐SLN 4%). Finally, the ZP values for all the empty formulations (approximately −30 mV) indicated good physical stability of the SLN dispersions.

**TABLE 2:** SLN physicochemical characterization: average diameter (Z‐AVE), polydispersity index (PI), zeta potential (ZP), and encapsulation efficiency (EE%) of freshly prepared empty SLN and CUR‐ or RSV‐loaded SLN prepared using different amounts of Compritol® 888 ATO (4%, 5%, and 6% wt/wt) and Transcutol® P (TRC; 0%, 2%, and 4% wt/wt)

SLN formulations	Z‐AVE (nm) ± *SD*	PI ± *SD*	ZP (mV) ± *SD*	EE% ± *SD*
Empty‐TRC 0%‐SLN 6%	162.2 ± 5.1	0.272 ± 0.005	−28.1 ± 6.2	—
Empty‐TRC 0%‐SLN 5%	140.4 ± 3.2	0.225 ± 0.017	−35.6 ± 2.4	—
Empty‐TRC 0%‐SLN 4%^a^	132.9 ± 3.8^a^	0.238 ± 0.010^a^	−35.5 ± 1.5^a^	—
Empty‐TRC 2%‐SLN 4%^a^	120.1 ± 13.8^a^	0.233 ± 0.009^a^	−35.2 ± 1.7^a^	—
Empty‐TRC 4%‐SLN 4%^a^	125.0 ± 5.5^a^	0.251 ± 0.018^a^	−30.3 ± 5.1^a^	—
CUR‐TRC 0%‐SLN 4%	149.0 ± 16.4	0.281 ± 0.006	−33.3 ± 1.8	96.7 ± 2.7
CUR‐TRC 2%‐SLN 4%	155.7 ± 16.7	0.339 ± 0.046	−34.0 ± 1.8	96.9 ± 2.7
CUR‐TRC 4%‐SLN 4%	158.8 ± 5.2	0.353 ± 0.081	−33.8 ± 2.9	99.0 ± 0.5
RSV‐TRC 0%‐SLN 4%	173.9 ± 15.4	0.329 ± 0.068	−36.8 ± 2.4	97.9 ± 1.5
RSV‐TRC 2%‐SLN 4%	158.6 ± 11.1	0.278 ± 0.026	−39.5 ± 2.1	98.3 ± 0.9
RSV‐TRC 4%‐SLN 4%	155.5 ± 5.0	0.276 ± 0.006	−38.7 ± 3.1	96.3 ± 2.6

*Note*: Mean values ± standard deviation (*SD*) obtained from at least three independent samples are reported.

Abbreviations: CUR, curcumin; RSV, resveratrol; SLN, solid lipid nanoparticles.

^a^ Data published in our previous work (Pitzanti et al., 2020).

Physicochemical characterization results on the obtained formulations showed that empty‐SLN with 2% and 4% of TRC exhibited a mean diameter that ranged between fairly narrow values (120–125 nm), which were not statistically different (*p* > 0.05) to that of starting formulation (empty‐TRC 0%‐SLN 4%). Also, PI and ZP seemed not to be directly influenced by TRC addition.

On the contrary, CUR and RSV loading (0.1 wt%) in the lipid matrix resulted in a significant increase of both size (values between 150 and 175 nm, *p* < 0.05) and PI values (0.28–0.35). The drug loading did not significantly affect the nanoparticle surface charge, which showed values close to that of empty SLN, ranging between −33 mV and −39 mV. As already highlighted before, nanoparticles with zeta potential values > +30 mV or < −30 mV are generally considered to have sufficient repulsive force to attain good physical colloidal stability during storage. The entrapment efficiency percentage of all CUR and RSV formulations was very high (EE% 97–99%) and independent of the TRC concentration (Table 2).

### 3.2 Role of lipid matrix concentration on SLN cytotoxicity and lipid profile modulation in 3T3 cells

In the first part of the study, empty SLN formulated with different amounts of the lipid matrix and without TRC (empty‐TRC 0%‐SLN 4%, empty‐TRC 0%‐SLN 5%, and empty‐TRC 0%‐SLN 6%) were tested in 3T3 fibroblasts for the evaluation of their impact on cell viability and lipid profile in relation to the lipid content.

Figure S1 shows the viability (expressed as percentage of the control) measured in control 3T3 fibroblasts (0) and cells treated for 2 h (Figure S1A) and 24 h (Figure S1B) with various aliquots (1.25, 2.5, and 5 μl/ml) of empty SLN prepared with different amounts of Compritol (4%, 5%, and 6%). The treatment with all empty SLN formulations induced, after 2 h‐incubation, a significant cytotoxic effect in 3T3 cells from the dose of 2.5 μl/ml, and a cell viability reduction, with respect to control cells, in the range 26–29% was observed at 5 μl/ml. However, SLN prepared with different amounts of Compritol® 888 ATO were not toxic in 3T3 cells at 24‐h incubation time (Figure S1B) and cell viability measured at the maximal tested dose (5 μl/ml) ranged from 94% to 98%. Starting from the consideration that the SLN cytotoxicity observed at short time of incubation (2 h) could be due to a variation in the cell redox status that affects mitochondrial metabolization of MTT (Falchi et al., 2015), a 24 h‐incubation time was chosen for successive studies in 3T3 cells.

**FIGURE 2:**
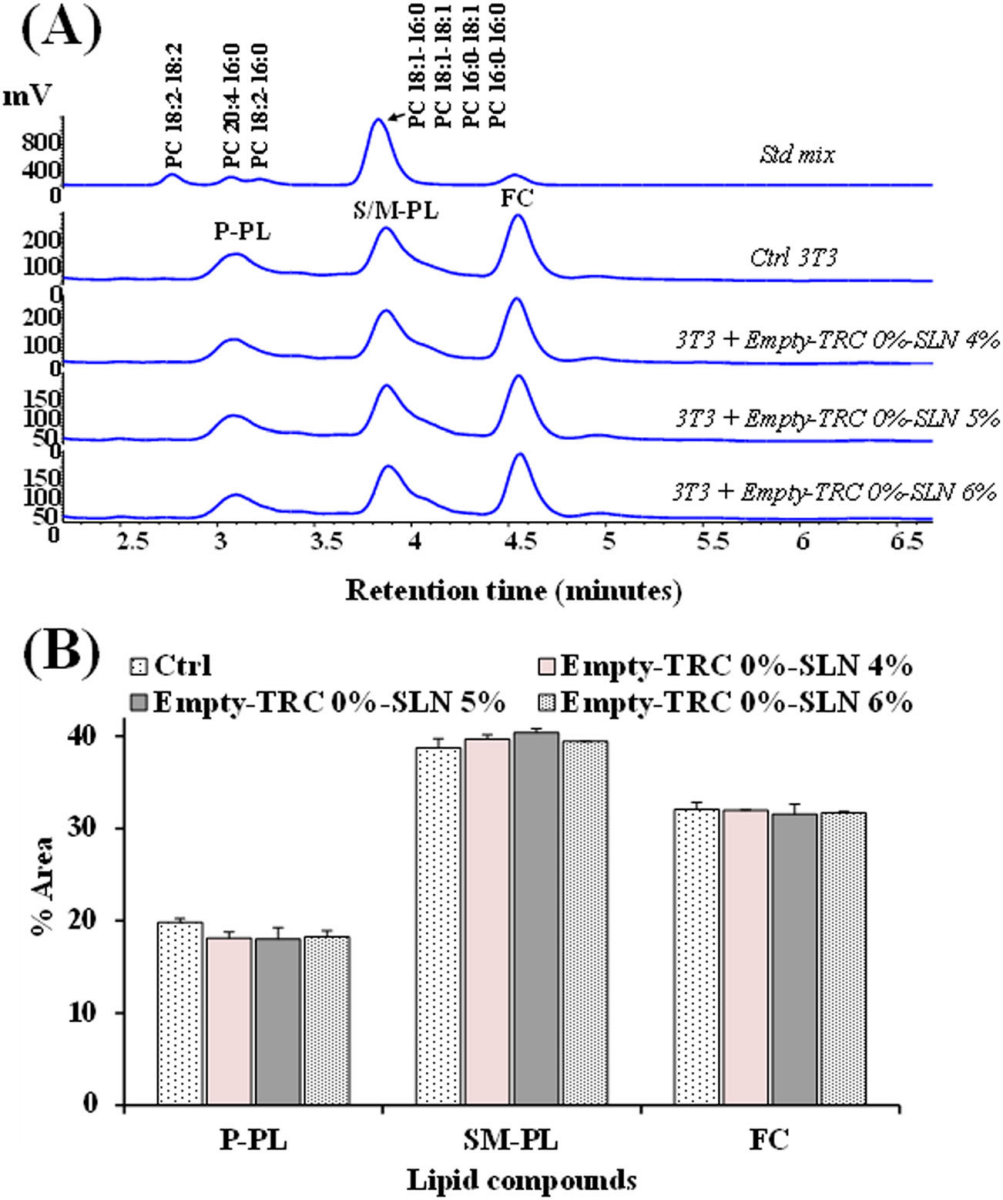
(A) Chromatographic profile, obtained by high‐performance liquid chromatography (HPLC)‐evaporative light scattering detector (ELSD) analysis, of saturated/monounsaturated phospholipids (S/M–PL), polyunsaturated phospholipids (P‐PL) and free cholesterol (FC), measured in control 3T3 cells (Ctrl) and cells treated for 24 h with 5 μl/ml of empty solid lipid nanoparticles (SLN) prepared with different amounts of Compritol® 888 ATO (4%, 5%, and 6%). The chromatographic region for each lipid class was assigned by using standard mixtures of saturated/monounsaturated (mix PL: PC 16:0/16:0, PC 18:1/18:1, PC 16:0/18:1, PC 18:1/16:0, ECN 32) and polyunsaturated phosphatidylcholines (PC 16:0/18:2, ECN 30; PC 16:0/20:4, PC 18:2/18:2, ECN 28; PC 20:5/20:5, ECN 20). (B) Values (percentage area) of S/M–PL, P–PL, and FC measured in control and SLN‐treated 3T3 fibroblasts. Results were expressed as a mean and standard deviation (*SD*) (*n* = 6). No significant differences were observed among samples (one‐way ANOVA and Bonferroni post hoc test). [Colour figure can be viewed at wileyonlinelibrary.com]

Unloaded SLN prepared with different amounts of lipid matrix were then tested in 3T3 cells to assess their effects after 24‐h incubation on cell lipid composition (main polar lipid classes and total FA profile). Figure 2 shows the chromatographic profiles (Figure 2A) and percentage area values (Figure 2B) of polar lipid compounds, mainly constituted by PL and FC, of 3T3 control cells and cells treated for 24 h with Compritol® 888 ATO (4%, 5%, and 6%) (at 5 μl/ml) obtained by HPLC‐ELSD analysis. Polar lipid profiles of control 3T3 were characterized by a peak of FC and two main peaks of PL, corresponding to saturated/monounsaturated PL (S/M‐PL) and polyunsaturated PL (P‐PL). Standard mixtures of phosphatidylcholines (S/M‐PL and P‐PL), and FC were used to assign the chromatographic region for each lipid class, as previously reported (Rosa et al., 2015, 2020). Cell polar lipids were separated on the basis of ECN (=*CN−2n*, where *CN* is the number of acyl group carbons and *n* is the number of double bonds) (Rosa et al., 2015, 2020). The 24‐h treatment of 3T3 cells with unloaded SLN prepared with different amounts of Compritol® 888 ATO (4%, 5%, and 6%) did not significantly affect cell lipid profile, independently from the lipid content. A similar slight decrease in the percentage amount of P‐PL (in the range 8.1–9.1%) and an increase in the percentage of S/M‐PL (in the range 1.9–4.3%) were observed in cell treated with unloaded SLN with respect to control cells.

**FIGURE 3:**
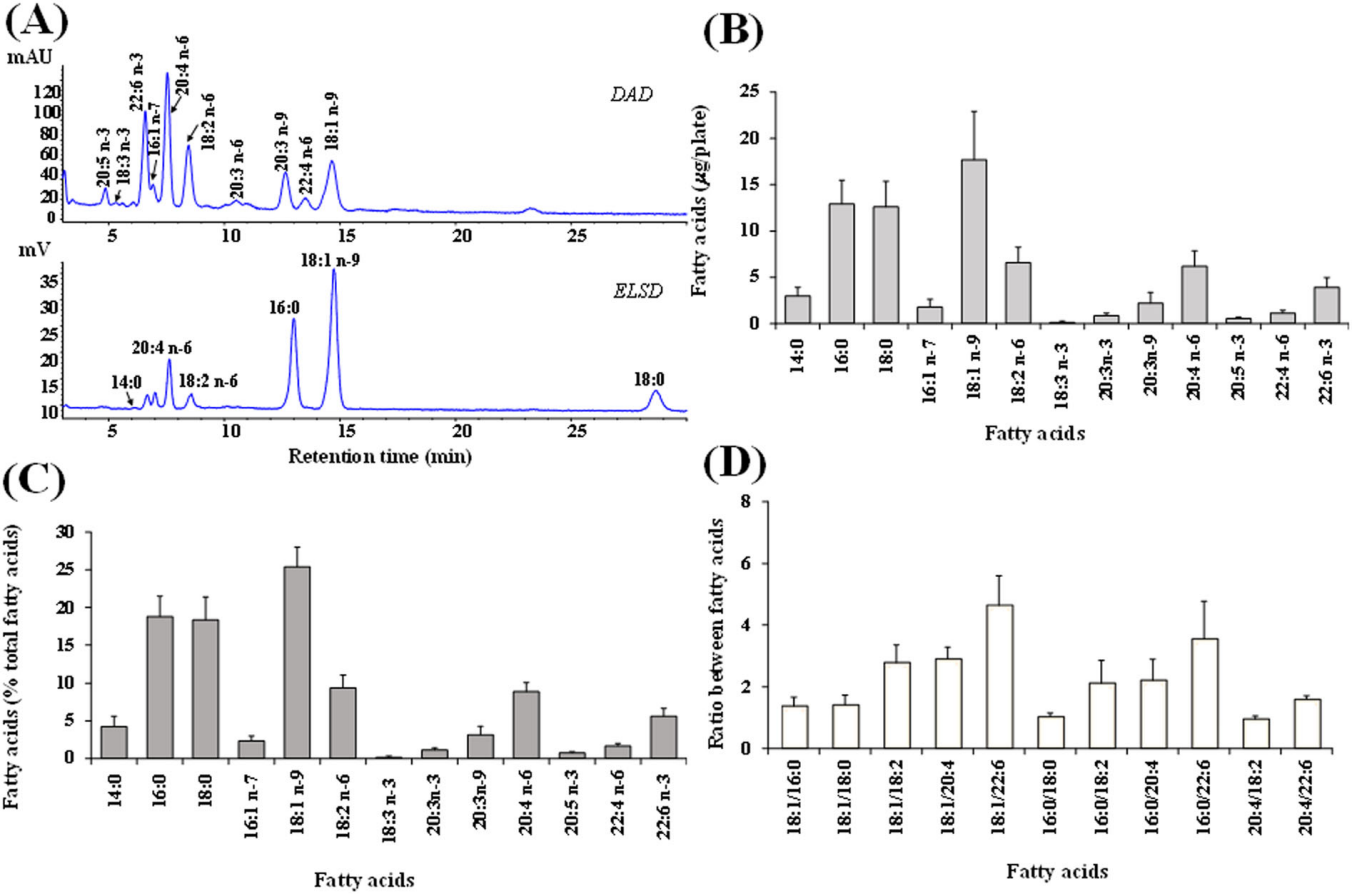
Chromatographic profile, obtained by high‐performance liquid chromatography (HPLC)‐ diode array detector (DAD)/evaporative light scattering detector (ELSD) (HPLC‐DAD/ELSD) analysis, of unsaturated (DAD) and saturated (ELSD) fatty acids (FA) measured in control 3T3 fibroblasts (A). Values of FA expressed as μg/plate (B) and percentage of total FA (C) measured in control 3T3 fibroblasts and values of the ratios among the main FA (D). Three independent experiments with two replicates for each condition are performed and data are presented as mean ± *SD* (*n* = 6). [Colour figure can be viewed at wileyonlinelibrary.com]

**FIGURE 4:**
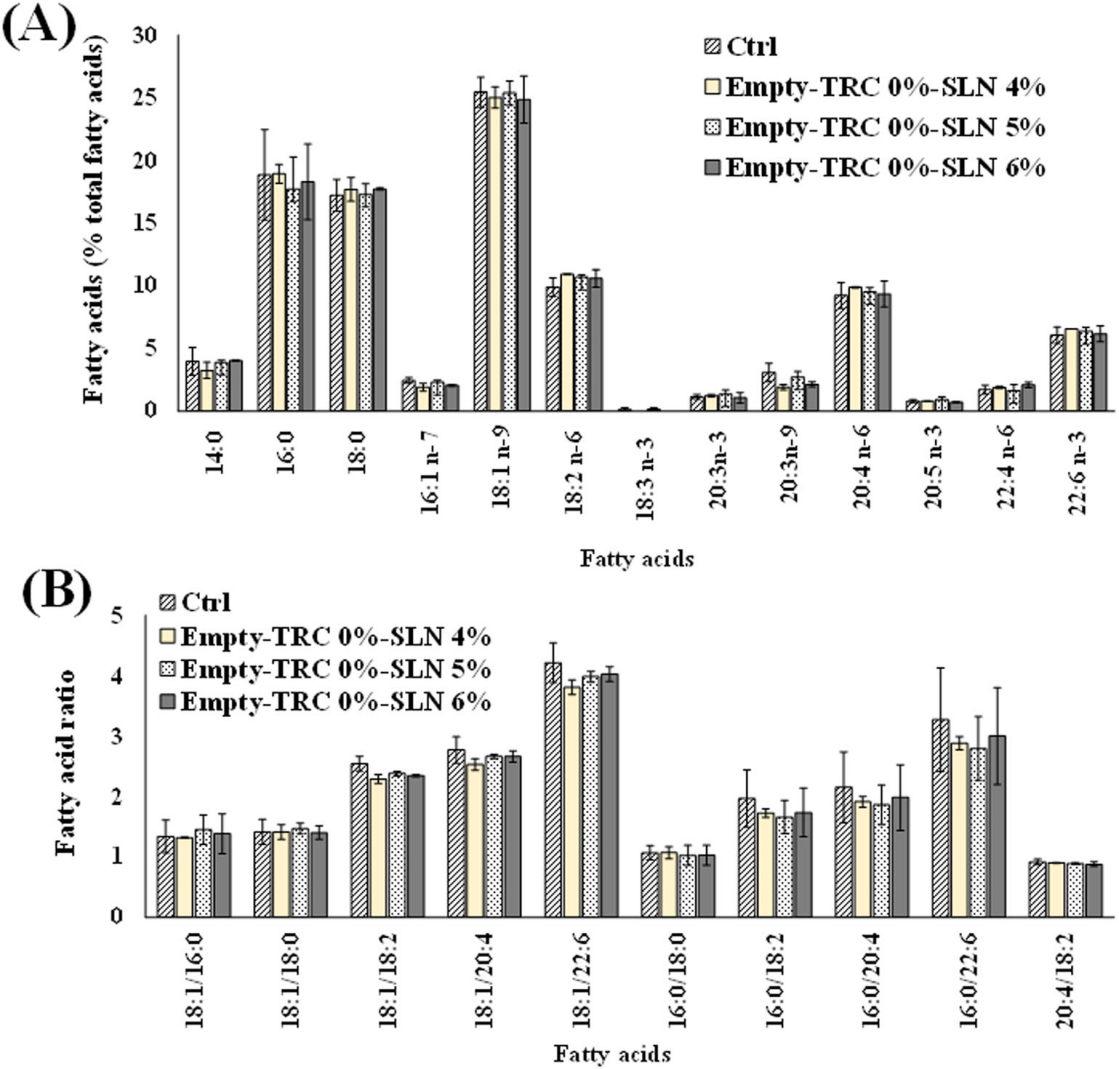
Values of the main fatty acids (FA) (expressed as percentage of total FA) (A) and their ratios (B) measured in control 3T3 fibroblasts (Ctrl) and cells treated for 24 h with 5 μl/ml of empty solid lipid nanoparticles (SLN) prepared with different amounts of Compritol® 888 ATO (4%, 5%, and 6%). Data are presented as mean ± *SD* (*n* = 6). No significant differences were observed among samples (one‐way ANOVA and Bonferroni post hoc test). [Colour figure can be viewed at wileyonlinelibrary.com]

Figure 3 shows the chromatographic profile, values (expressed as μg/plate and percentage of total FA) of unsaturated and saturated FA, and their ratios measured in control 3T3 cells. Control fibroblasts showed an FA composition characterized by a high level of oleic acid 18:1 n‐9 (17.7 ± 5.2 μg/plate, 25.4% of total FA), palmitic acid 16:0 (12.9 ± 2.6 μg/plate, 18.8%), and stearic acid 18:0 (12.6 ± 2.8 μg/plate, 18.4%), while linoleic acid 18:2 n‐6 (6.6 ± 1.7 μg/plate, 9.4%) and arachidonic acid 20:4 n‐6 (6.2 ± 1.7 μg/plate, 8.9%) represented the most abundant FA among polyunsaturated FA (PUFA), followed by docosahexaenoic acid 22:6 n‐3 (Figure 3B,C). Values of ratios among main FA determined in 3T3 cells were 8.4 ± 3.4, 4.6 ± 0.9, 3.6 ± 1.2, 2.9 ± 0.4, 2.8 ± 0.6, and 1.6 ± 0.1 for 16:0/16:1 n‐7, 18:1/22:6 n‐3, 16:0/22:6 n‐3, 18:1 n‐9/20:4 n‐6, 18:1 n‐9/20:4 n‐6, 18:1 n‐9/18:2 n‐6, and 20:4 n‐6/22:6 n‐3, respectively (Figure 3D). The incubation of fibroblasts with SLN prepared with different amounts of Compritol® 888 ATO (empty‐TRC 0%‐SLN 4%, empty‐TRC 0%‐SLN 5% and empty‐TRC 0%‐SLN 6%) did not induced marked changes in the FA amounts (Figure 4A) and their ratios (Figure 4B) with respect to untreated‐control cells.

### 3.3 Effect of SLN formulations loaded with CUR and RSV on 3T3 cell viability and lipid profile

The increase in the percentage amount of the lipid matrix did not induce a significant change in 3T3 cell viability and lipid profile; therefore, the empty‐TRC 0%‐SLN 4% was then selected as starting formulation, subsequently modified by adding different TRC percentages (Pitzanti et al., 2020) and loading the two bioactive liposoluble polyphenolic compounds CUR and RSV (Figure 1). The concentration of 0.1% for CUR and RSV was chosen on the basis of a preliminary screening conducted to find the concentration of polyphenols that assured the highest encapsulation efficiency and our previous study performed on 8‐methoxypsoralen‐SLN loaded (Pitzanti et al., 2020).

**FIGURE 5:**
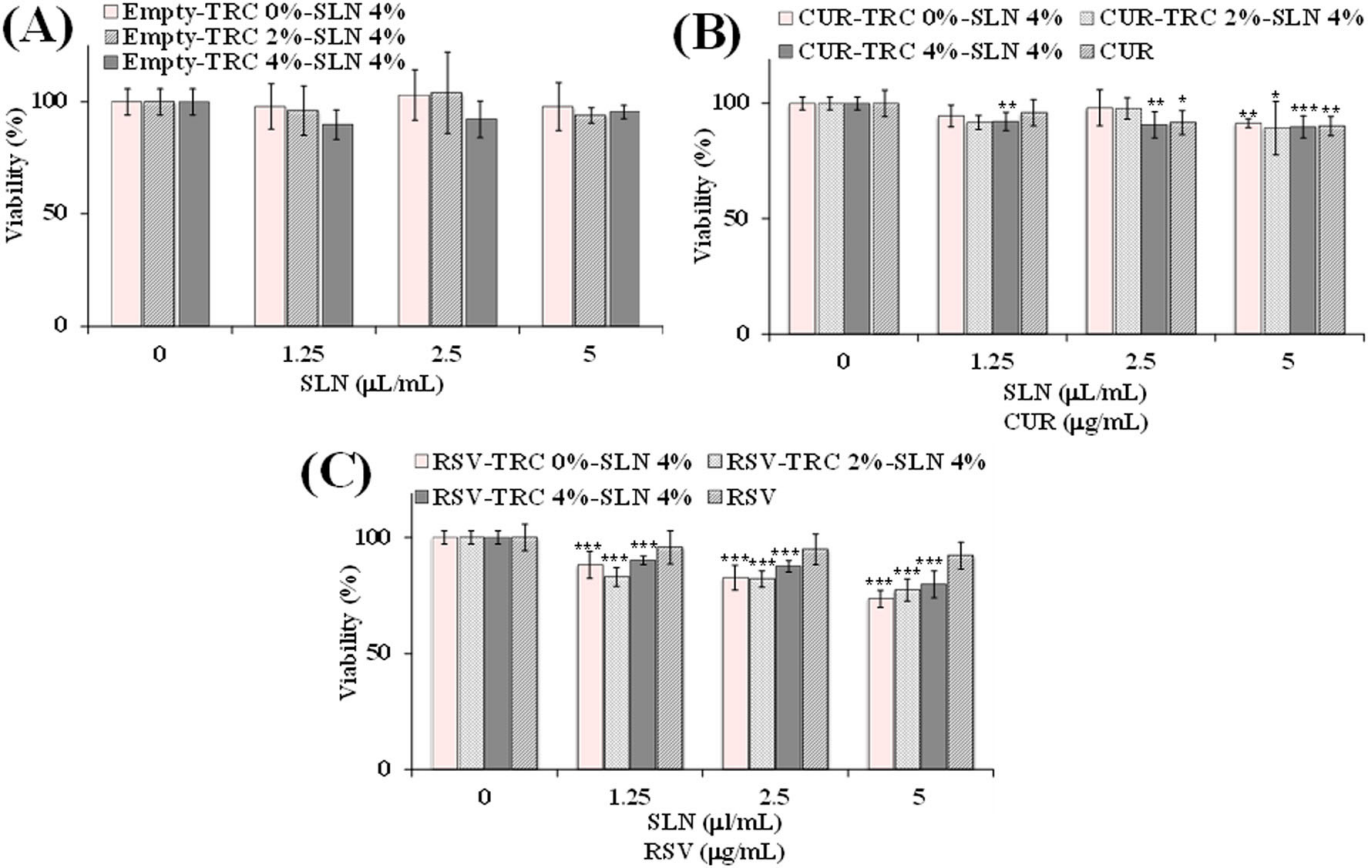
Viability (expressed as percentage of the control) (MTT assay) measured in control 3T3 fibroblasts (0) and cells treated for 24 h with different aliquots (1.25, 2.5, 5 μl/ml) of unloaded solid lipid nanoparticles (SLN) 4% (with TRC 0%, 2%, and 4%) (A), curcumin (CUR)‐loaded SLN 4% (with TRC 0%, 2%, and 4%) (B) and resveratrol (RSV)‐loaded SLN 4% (with TRC 0%, 2%, and 4%) (C). Treatment with pure compounds CUR and RSV (1.25, 2.5, 5 μg/ml) is also reported. Data are mean ± standard deviation (*SD*) (*n* = 12); ***p < 0.001, **p < 0.01, *p < 0.05 versus control (one‐way ANOVA and Bonferroni post hoc test). [Colour figure can be viewed at wileyonlinelibrary.com]

Figure 5 shows the viability (expressed as percentage of the control) (MTT assay) measured in control 3T3 fibroblasts and cells treated for 24 h with different aliquots (1.25, 2.5, and 5 μl/ml) of empty and CUR‐ and RSV‐loaded SLN 4% (with TRC 0%, 2%, and 4%). Treatment with pure compounds CUR and RSV, tested at the same dose present in SLN, is also reported. No significant changes in cell viability, with respect to control cells, were observed in 3T3 fibroblasts treated with unloaded SLN with TRC (TRC 2%‐SLN 4% TRC 4%‐SLN 4%) (Figure 5A). CUR‐loaded SLN with different amounts of TRC (0%, 2%, and 4%) (Figure 5B) did not show a marked toxic effect on 3T3 cells; however, a viability reduction in the range 5–10% was observed at the different tested concentrations. A statistically significant viability reduction (≤10%) was observed for all CUR‐loaded SLN at 5 μl/ml; however, percentages of cell viability above 90% are generally considered as noncytotoxicity. RSV‐loaded SLN with 0%, 2%, and 4% of TRC (Figure 5C) showed a certain cytotoxic effect, higher than other SLN and pure RSV. Reduction of viability (in the range 21–27% at 5 μl/ml) was similar in cells treated with all types of RSV‐loaded SLN, independently from TRC amount. Therefore, unloaded and loaded SLN with 4% TRC were chosen for the evaluation of the impact on lipid profile.

**FIGURE 6:**
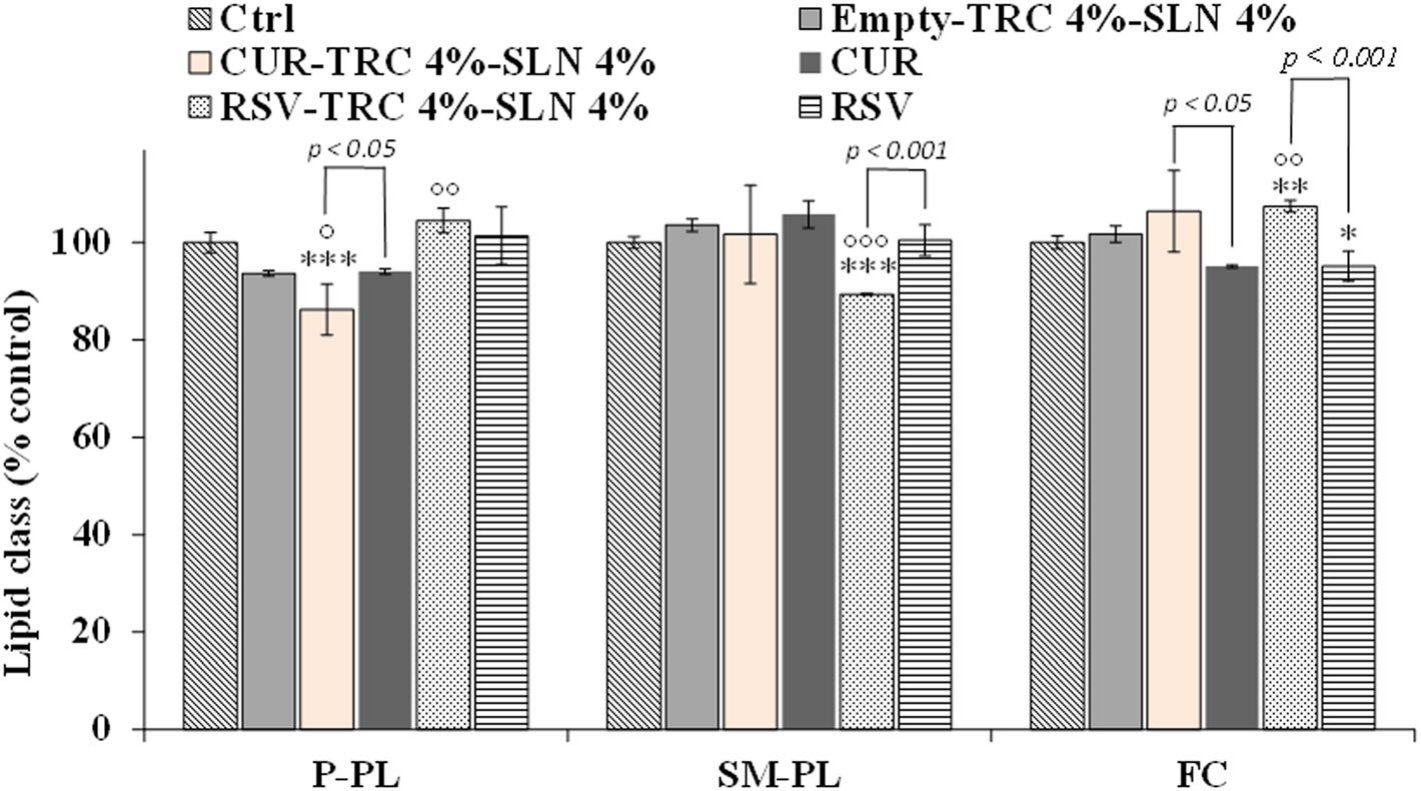
Values (expressed as percentage control) of saturated/monounsaturated phospholipids (S/M–PL), polyunsaturated phospholipids (P‐PL) and free cholesterol (FC), measured in control 3T3 cells (Ctrl) and cells treated for 24 h with 5 μl/ml of unloaded (empty‐TRC 4%‐SLN 4%) and loaded SLN (curcumin [CUR]‐TRC 4%‐SLN 4% and resveratrol [RSV]‐TRC 4%‐SLN 4%). Results were expressed as a mean and standard deviation (*SD*) (*n* = 6). Treatment with pure compounds (CUR and RSV) is also reported. Data are mean ± standard deviation (*SD*) (*n* = 6); ***p < 0.001, **p < 0.01, *p < 0.05 versus control; °°°*p* < 0.001, °°*p* < 0.01, °*p* < 0.05 versus unloaded SLN (one‐way ANOVA and Bonferroni post hoc test). SLN, solid lipid nanoparticles. [Colour figure can be viewed at wileyonlinelibrary.com]

Figure 6 shows the values (expressed as percentage control) of the different classes of phospholipids (S/M–PL, P‐PL) and FC measured in control 3T3 cells and cells treated for 24 h with 5 μl/ml of unloaded empty‐(TRC 4%‐SLN 4%) and loaded SLN (CUR‐TRC 4%‐SLN 4% and RSV‐TRC 4%‐SLN 4%). Treatment with pure CUR and RSV is also reported. The 24‐h treatment of 3T3 cells with unloaded SLN (empty‐TRC 4%‐SLN 4%) affected polar lipid profile with respect to control cells, inducing an evident, unless not significant, decrease in the percentage amount of P‐PL and an increase in the percentage of S/M‐PL. SLN loaded with CUR (CUR‐TRC 4%‐SLN 4%) and RSV (RSV‐TRC 4%‐SLN 4%) modulated 3T3 cell lipid profile differently with respect to unloaded SLN. CUR‐loaded SLN induced a more marked decrease in the percentage amount of P‐PL than unloaded SLN, contemporarily increasing the percentage of FC. Cell treated with RSV‐loaded SLN induced, with respect to unloaded SLN, a significant increase in the percentage amount of P‐PL, coupled to a significant decrease in the percentage level of S/M‐PL and a marked increase in the amount of FC. Cell treatment with pure CUR affected lipid profile with respect to control cells, with a slight decrease in the percentage levels of P‐PL and FC, together with a SM‐PL increase. Pure RSV slightly affected cell lipid profile, and RSV‐treated cells showed a lipid profile very similar to that of control cells.

**FIGURE 7:**
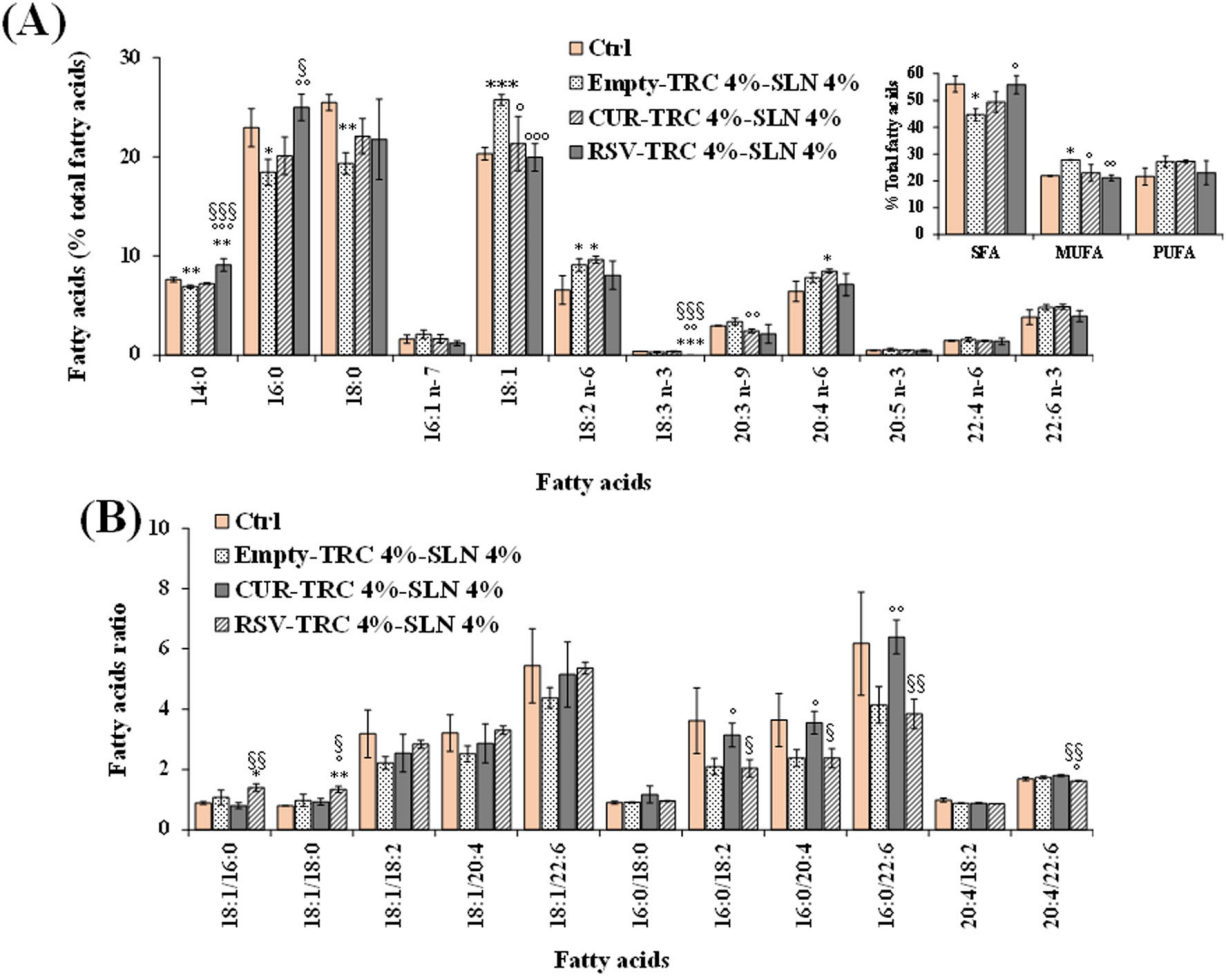
Values of the main saturated (SFA), monounsaturated (MUFA) and polyunsaturated (PUFA) fatty acids (FA) (expressed as percentage of total FA) (A) and their ratios (B) measured in control 3T3 fibroblasts (Ctrl) and cells treated for 24 h with 5 μl/ml of unloaded (empty‐TRC 4%‐SLN 4%) and loaded SLN (CUR‐TRC 4%‐SLN 4% and RSV‐TRC 4%‐SLN 4%). Data are mean ± standard deviation (*SD*) (*n* = 6); ***p < 0.001, **p < 0.01, *p < 0.05 versus control; °°°*p* < 0.001, °°*p* < 0.01, °*p* < 0.05 versus unloaded SLN; ^§§§^ *p* < 0.001, ^§§^ *p* < 0.01, ^§^ *p* < 0.05 versus CUR‐TRC 4%‐SLN 4% (one‐way ANOVA and Bonferroni post hoc test). [Colour figure can be viewed at wileyonlinelibrary.com]

Values of the main FA (expressed as percentage of total FA) and their ratios measured in control 3T3 fibroblasts and cells treated for 24 h with 5 μl/ml of unloaded (empty‐TRC 4%‐SLN 4%) and loaded SLN (CUR‐TRC 4%‐SLN 4% and RSV‐TRC 4%‐SLN 4%) are shown in Figure 7. The 24‐h treatment of 3T3 cells with unloaded SLN (empty‐TRC 4%‐SLN 4%) affected FA profile with respect to control cells (Figure 7A), inducing a remarkable significant decrease in the percentage cell level of saturated FA (SFA) with respect to control cells, evident for 16:0 (*p* < 0.05) and 18:0 (*p* < 0.01), coupled to a significant increase in the level of monounsaturated FA (MUFA), manly represented by 18:1 n‐9 (*p* < 0.001 vs. untreated cells), and *to a minor extent*, polyunsaturated FA (PUFA). Differences with respect to control cells were also observed in the values of FA ratios (Figure 7B). The loading of SLN with CUR and RSV significantly modulated the impact of nanoparticles on cells. An evident, but not significant, decrease of SFA (16:0 and 18:0) was also observed in CUR‐loaded SLN, coupled to a small increase in the PUFA level (mainly 20:4 n‐6 and 18:2 n‐6). Significant changes, with respect to unloaded SLN, were observed for 16:0/18:2 n‐6, 16:0/20:4 n‐6 and 16:0/22:6 n‐3 ratios. Cells treated with RSV‐TRC 4%‐SLN 4% showed an FA profile significantly different from that of fibroblasts treated with unloaded SLN and similar to control cells. However, some differences were observed in the FA ratios (16:0/18/2 n‐6, 16:0/20:4‐n‐6, and 16:0/22:6 n‐3) with respect to control cells, with values similar to unloaded SLN‐treated cells, confirming the modulatory effect of RSV‐loaded SLN on lipid components.

A preliminary study was performed to determine the CUR absorption in 3T3 cells. Figure S2 shows the chromatographic profiles and UV spectra (at the wavelength of 450 nm) of peak measured in the chloroform fractions obtained from control 3T3 cells and cells treated for 24 h with 0.5 μg/ml of pure CUR and 0.5 μl/ml of CUR‐TRC 4%‐SLN 4%. The presence of the phenol in cell extract was assessed by comparison with the retention time and spectrum of the CUR standard. 3T3 cells treated with pure CUR did not show, in our experimental condition, the compound in cell pellets, while a CUR value of 6.0 ± 2.3 μg/plate was measured in the pellet of 3T3 fibroblasts treated with CUR‐loaded SLN (Figure S2).

## 4 DISCUSSION

SLN can be used to deliver drugs orally, topically, or via inhalation (Faraji & Wipf, 2009; Battaglia & Ugazio, 2019). The knowledge of SLN interaction with living systems is essential in the perspective of implementing nanotechnologies in a safe way (Severino et al., 2012). LN are characterized by a high stability and ability to carry hydrophilic and lipophilic compounds in the target organ and are generally well tolerated by the human body, because they are made from physiological compounds leading to the metabolic pathways, minimizing adverse side effects (Musielak et al., 2022; Severino et al., 2012). In this study, several empty and CUR‐ or RSV‐loaded SLN formulations were physiochemically characterized and tested in 3T3 murine fibroblasts for the evaluation of their impact on cell viability and lipid profile in relation to the lipid content and incorporated compound.

All the empty formulations did not show cytotoxic effect in 3T3 cells at long‐term exposure (24 h of incubation), independently of the lipid and TRC amount; therefore, this incubation time was chosen for all cell viability assessments. Components (Compritol®, TRC and Poloxamer 188) of empty SLN are safe and well‐tolerated compounds, used in many cosmetic and pharmaceutical products for skin and/or oral delivery (Björklund et al., 2016; Cortés et al., 2021; Devi & Agarwal, 2019).

In our experimental conditions, CUR and RSV loading in the lipid matrix determined an increase of SLN size and PI values, without significant effects on the nanoparticle surface charge. EE% values of 97–99% were measured for all CUR and RSV formulations, and it is reasonable to believe that this high loading capacity is due to both the high hydrophobicity of the loaded molecules and the imperfect crystalline structure produced by Compritol® during re‐crystallization, which reduces drug expulsion from the lipid matrix. (Müller et al., 2000; Righeschi et al., 2016). The results obtained in this work confirmed that the higher encapsulation efficiency and long‐term physical stability of the SLN have been assured by the following component composition: Compritol 888 ATO 4%, Transcutol 0–4%, Poloxamer 188 2.2%, and drug 0.1%. (Pitzanti et al., 2020).

All CUR‐loaded SLN formulated with different amounts of TRC (0%, 2%, and 4%) showed a slight toxic effect on 3T3 fibroblasts, with a statistically significant viability reduction (≤10%) observed at the highest tested dose (5 μl/ml). Previous study showed minimal cytotoxicity of a series of CUR‐loaded solid lipid‐polymer hybrid nanoparticles (SLPN) in human epithelial Caco‐2 cells, used as model to study the CUR cellular uptake (Wang et al., 2018). RSV loading significantly improved SLN cytotoxic effect, independently from the TRC amount, with a viability reduction in the range 21–27% at the highest tested dose. Some toxicity was previously reported for RSV‐loaded SLN and nanostructured lipid carriers in an immortalized cell line of human keratinocytes (HaCaT) (Rocha et al., 2017).

Empty SLN without TRC did not affect phospholipids and FA profile of treated 3T3 cells, which showed values like control cells, indicating a very slight ability of SLN to interact with cell membranes, independently from the lipid matrix amount. The incorporation of TRC into SLN (empty‐TRC 4%‐SLN 4%) increased their capacity to interact with cells, modulating phospholipids and FA profile of fibroblasts with respect to control cells after 24‐h incubation, without affecting cell viability. TRC is a penetration enhancer, a safe and well‐tolerated solvent/vehicle used in the formulation or manufacturing process of pharmaceuticals, cosmetics, and food additives (Björklund et al., 2016; Sullivan et al., 2014). The observed significant increase in the cell level of S/M‐PL and MUFA, mainly 18:1 n‐9, after the treatment with empty‐TRC 4%‐SLN 4% could be probably ascribable to an enhanced cellular uptake of nanoparticles (Panariti et al., 2012) helped by TRC, coupled to the metabolism of behenic acid (22:0), the main FA of Compritol® (Devi & Agarwal, 2019). After SLN membrane interaction/uptake, behenic acid, derived from the lipolysis of its glyceryl derivatives, was possibly metabolized/oxidized by fibroblasts into the shorter‐chain FA 18:1 n‐9, which was then used for the synthesis of phospholipids. A previous study reported the cellular uptake of SLN composed of Compritol® 888ATO as lipid material (Yuan et al., 2008). In humans, dietary behenic acid is poorly absorbed because of its low bioavailability compared with other FA (Cater & Denke, 2001); therefore, the modulation of lipid profile observed in 3T3 cells after treatment with empty‐TRC 4%‐SLN 4% is a clear indication of SLN uptake/interaction with plasma membrane (Panariti et al., 2012).

The loading of SLN with CUR and RSV significantly modulated the impact of nanoparticles on cell lipids with respect to empty SLN. CUR‐TRC 4%‐SLN 4% induced a noticeable increase in FC cell level, a less marked decrease of SFA and increase of 18:1 n‐9 than empty‐TRC 4%‐SLN 4%, coupled to a small increase in the PUFA level. CUR is a hydrophobic compound, and previous studies evidenced its ability to accumulate in cell membranes (partition into lipid bilayers), leading to an alteration of the membrane environment and particular membrane properties as the fluidity and the phase transition temperature (Duda et al., 2020; Leite et al., 2018). CUR acts through the membranes by changing the general lipid membrane organization and exerts its antitumor activity in cancer cells by inhibiting the expression of enzymes involved in lipid metabolism like FA synthase and stearoyl‐CoA desaturase (Naeini et al., 2019). In this study, we assessed the modulatory effect on lipid profile of CUR‐loaded SLN at the concentration of 5 μl/ml approximately corresponding, considering the EE% value of 97–99%, to CUR 13 μM. Previous study demonstrated that at concentrations of the order of 100 μM, CUR exhibits destabilization effects on membranes, but at much lower concentrations it modulates the function of membrane proteins (Leite et al., 2018). Taking into consideration the differences observed between the effect on lipid profile of empty‐TRC 4%‐SLN 4% and CUR‐TRC 4%‐SLN 4%, the modulatory effect on the lipid components observed in 3T3 fibroblasts treated with CUR‐loaded SLN was possibly the result of the synergistic/combined impact of the lipid matrix and pure CUR on cell lipid metabolism. Previous studies evidenced synergistic effects of nanostructured lipid carriers with the encapsulated compound (Beloqui et al., 2016; Huguet‐Casquero et al., 2020). The presence of CUR in the cell pellets of 3T3 fibroblasts treated with CUR‐TRC 4%‐SLN 4%, corresponding approximately to 12% of the applied amount, confirmed the uptake of CUR‐loaded SLN and the interaction/penetration of the phenol in cell membranes whereas in our experimental conditions, CUR was not detected in cells treated with the pure compound.

Also, the treatment of 3T3 cells with RSV‐loaded SLN modulated PL and FA profile differently from unloaded and CUR‐loaded SLN. Cell treatment with RSV‐TRC 4%‐SLN 4% induced, with respect to unloaded SLN, a significant increase in the amount of P‐PL and FC, together to a decrease in the S/M‐PL level. Cells treated with RSV‐TRC 4%‐SLN 4% showed an FA profile more similar to control cells than unloaded SLN‐treated cells; however, some differences were observed in the profile of FA ratios versus controls, confirming the modulatory effect of RSV‐loaded SLN on lipid components. FA ratios data have been indicated as a suitable data set that effectively replaces the original data set for a quantitative comparative evaluation of cell FA composition, being irrespective of the number of FA included in the studies and the units of quantitative measurement (Graeve & Greenacre, 2020). In our experimental conditions, the 5 μl/ml concentration of RSV‐loaded SLN used for lipid profile modulation corresponded, considering the EE% of 97–99%, approximately to an RSV concentration of 21 μM. A previous study demonstrated the impact of RSV on FA metabolism in cultured hepatocytes (Kühn et al., 2018). Incubation of human HepG2 cells with RSV 40 μM induced a significant reduction in the levels of MUFA (16:1 n‐7, 18:1 n‐9, 18:1 n‐7, and 20:1 n‐9), coupled to an increase in SFA levels (mainly stearic acid), due to a modulatory activity on the expression of enzymes involved in lipid metabolism like desaturases (Kühn et al., 2018). Moreover, RSV showed the ability to modify PL and FA composition in cancer cell lines after 24‐h treatment, at the dose of 80 and 200 μM in MCF‐7 and MDA‐MB‐231 cells, respectively (Gomes et al., 2020). As observed for CUR‐loaded SLN, the effect of RSV‐loaded SLN on 3T3 cell lipid profile was maybe the consequence of the synergistic impact (Beloqui et al., 2016; Huguet‐Casquero et al., 2020) of the lipid matrix and pure RSV on cell lipid metabolism, taking into consideration the difference observed in the lipid modulatory effect with respect to the empty‐TRC 4%‐SLN 4%.

Taken together, our results showed the impact of CUR‐loaded and RSV‐loaded SLN on lipid metabolism, with a slight toxic effect on cell viability, more marked in the presence of RVS as encapsulated drug. The PL and FA modification observed in 3T3 cells after SLN treatment is physicochemically relevant. Alterations in cell membrane lipid composition, like changes in the ratio between SFA and MUFA chains of the PL, can directly influence the biophysical properties of biological membranes, deeply altering fluidity, the lipid raft thermodynamic properties, and microdomain organization (Hagen et al., 2010). Changes in lipid composition influence many cellular functions such as signal transduction, cell adhesion, lipid trafficking, ion channel function, receptor mobility/interaction, and the biological response to the drugs (Hagen et al., 2010). Therefore, the evaluation of the modulatory effect on cell lipids is essential in the study of biocompatibility of new SLN formulations. In addition, SLN formulated with a specific combination of the lipid matrix and loaded compound could represent a potential platform to target lipid metabolism in those pathological conditions characterized by altered lipid metabolic pathways.

## 5 CONCLUSION

SLN are generally considered nontoxic and highly compatible but could affect cell viability and lipid profile. In this study, we demonstrated the impact of CUR‐ and RSV‐loaded SLN on lipid metabolism (cell polar lipids and fatty acid profile), with a slight toxic effect on cell viability. The incorporation of TRC into SLN (empty‐TRC 4%‐SLN 4%) increased their capacity to interact with cells. Empty‐TRC 4%‐SLN 4%, CUR‐TRC 4%‐SLN 4%, and RSV‐TRC 4%‐SLN 4% showed the capacity to modify the lipid profile/metabolism of 3T3 cells due to the SLN uptake; however, this effect changed in relation to the encapsulated compound. The lipid modulatory effect of SLN in cells has important biochemical and physiological implications because changes in the cell lipid composition can influence biological membrane properties and affect several cellular processes. Our results highlighted how the combined impact on lipid metabolism of SLN and loaded drug should be taken into consideration in the evaluation of toxicity, potential application, and therapeutic effects of new formulations. This study represents an initial step for future studies that could explore more in depth the dose–response relationship of CUR‐loaded‐ and RSV‐loaded SLN on lipid profile modulation for the assessment of their biocompatibility and potential therapeutic application in lipid disorders.

## CONFLICTS OF INTEREST

The authors declare that they have no conflict of interest.

## Supporting information


**Table S1.** Calibration curves for fatty acid quantification in 3T3 cells.
**Figure S1.** Viability (expressed as % of the control) (MTT assay) measured in control 3T3 fibroblasts (0) and cells treated for 2 h (**A**) and 24 h (**B**) with aliquots (1.25, 2.5, 5 μL/mL) of empty SLN prepared with different amount of Compritol® 888 ATO (4, 5, and 6%). Data are presented as mean ± standard deviation (SD) (n = 12). of three independent experiments; *** = p < 0.001, ** = p < 0.01, * = p < 0.05 versus Ctrl (One–way ANOVA and Bonferroni post hoc Test).
**Figure S2:** Chromatographic profiles and UV spectra (450 nm) of peaks measured in the chloroform fractions obtained from control 3T3 cells and cells treated with pure CUR (5 μg/mL) and CUR‐TRC 4%‐SLN 4% (5 μL/mL). CUR in cell extracts was identified by comparison with the retention time and spectrum of CUR standard.

## Data Availability

The data that support the findings of this study are available from the corresponding author upon reasonable request.
