# Supplementary material for: Impact of solid lipid nanoparticles on 3T3 fibroblasts viability and lipid profile: The effect of curcumin and resveratrol loading
Source: J Appl Toxicol. 2022 Aug 26;43(2):272–86. doi: 10.1002/jat.4379 (PMC10087382; doi:10.1002/jat.4379)
Supplement: Supplementary file 1 — Table S1. Calibration curves for fatty acid quantification in 3T3 cells. Figure S1. Viability (expressed as % of the control) (MTT assay) measured in control 3T3 fibroblasts (0) and cells treated for 2 h (A) and 24 h (B) with aliquots (1.25, 2.5, 5 μL/mL) of empty SLN prepared with different amount of Compritol® 888 ATO (4, 5, and 6%). Data are presented as mean ± standard deviation (SD) (n = 12). of three independent experiments; *** = p < 0.001, ** = p < 0.01, * = p < 0.05 versus Ctrl (One–way ANOVA and Bonferroni post hoc Test). Figure S2: Chromatographic profiles and UV spectra (450 nm) of peaks measured in the chloroform fractions obtained from control 3T3 cells and cells treated with pure CUR (5 μg/mL) and CUR‐TRC 4%‐SLN 4% (5 μL/mL). CUR in cell extracts was identified by comparison with the retention time and spectrum of CUR standard. [file JAT-43-272-s001.docx]

Supporting Information

Impact of solid lipid nanoparticles on 3T3 fibroblasts viability and lipid profile: effect of curcumin- and resveratrol-loading

Antonella Rosa^1,^*, Mariella Nieddu^1^, Giulia Pitzanti^2^, Rosa Pireddu^2^, Francesco Lai^2^, and Maria Cristina Cardia^2^

|  |
| --- |

^1^ Department of Biomedical Science, University of Cagliari, 09042 Monserrato, Cagliari, Italy; [anrosa@unica.it](mailto:anrosa@unica.it) (A.R.); [mnieddu@unica.it](mailto:mnieddu@unica.it) (M.N.)

^2^ Department of Life and Environmental Sciences, University of Cagliari, Via Ospedale, 72, 09124, Cagliari, Italy; [giulia.pitzanti@unica.it](mailto:giulia.pitzanti@unica.it) (G.P.); [rosapireddu@unica.it](mailto:rosapireddu@unica.it) (R.P.); [frlai@unica.it](mailto:frlai@unica.it) (F.L.); [cardiamr@unica.it](mailto:cardiamr@unica.it) (M.C.C.)

*****Correspondence: [anrosa@unica.it](mailto:anrosa@unica.it); Tel.: +39 675 4124

**Table S1.** Calibration curves for fatty acid quantification in 3T3 cells.

| Fatty acid | Mass o.c. (ng) | Calibration curve equation | Correlation coefficient (R^2^) | LOD (ng o.c.) |
| --- | --- | --- | --- | --- |
| *ELSD detection (quadratic curve)* | | | | |
| 14:0 | 500-3500 | y = 31.79x^2^ – 68.088x + 73.757 | 0.9950 | 100 |
| 16:0 | 1000-3500 | y = 11.834x^2^ + 160.12x - 0.81 | 0.9909 | 100 |
| 18:0 | 500-3000 | y = 9.2768x^2^ + 0.4854x + 21.97 | 0.9995 | 150 |
| *DAD detection at 200 nm (linear curve)* | | | | |
| 16:1 n-7 | 1000-10000 | y = 849.09x – 575.16 | 0.9768 | 50 |
| 18:1 n-9 | 1000-10000 | y = 548.06x – 332.64 | 0.9705 | 100 |
| 18:2 n-6 | 500-6000 | y = 1080.7x – 742.11 | 0.9953 | 25 |
| 18:3 n-3 | 500-6000 | y = 1142.4x – 808.03 | 0.9835 | 10 |
| 20:3 n-6 | 500-6000 | y = 1364.9x – 885.0 | 0.9952 | 25 |
| 20:4 n-6 | 500-6000 | y = 1951.4x – 1075.7 | 0.9973 | 10 |
| 20:5 n-3 | 500-6000 | y = 2338.4x – 1315.7 | 0.9960 | 5 |
| 22:4 n-6 | 500-6000 | y = 1914,2x – 1781.3 | 0,9714 | 10 |
| 22:6 n-3 | 500-6000 | y = 2233.1x – 1005.6 | 0.9961 | 5 |

*Abbreviation*: o.c., on column; LOD, Limit of Detection. 20:3 n-9 was quantified as 20:3 n-6

|  |
| --- |

**FIGURE S1.** Viability (expressed as % of the control) (MTT assay) measured in control 3T3 fibroblasts (0) and cells treated for 2 h (**A**) and 24 h (**B**) with aliquots (1.25, 2.5, 5 μL/mL) of empty SLN prepared with different amount of Compritol® 888 ATO (4, 5, and 6%). Data are presented as mean ± standard deviation (SD) (n = 12). of three independent experiments; *** = p < 0.001, ** = p < 0.01, * = p < 0.05 versus Ctrl (One–way ANOVA and Bonferroni post hoc Test).

**FIGURE S2:** Chromatographic profiles and UV spectra (450 nm) of peaks measured in the chloroform fractions obtained from control 3T3 cells and cells treated with pure CUR (5 μg/mL) and CUR-TRC 4%-SLN 4% (5 μL/mL). CUR in cell extracts was identified by comparison with the retention time and spectrum of CUR standard.
